# Fluorescent Lipids: Functional Parts of Fusogenic Liposomes and Tools for Cell Membrane Labeling and Visualization

**DOI:** 10.3390/molecules17011055

**Published:** 2012-01-20

**Authors:** Christian Kleusch, Nils Hersch, Bernd Hoffmann, Rudolf Merkel, Agnes Csiszár

**Affiliations:** Institute of Complex Systems, ICS-7: Biomechanics, Forschungszentrum Jülich GmbH, Jülich 52425, Germany

**Keywords:** fusogenic liposomes, cellular membrane staining, fluorescent lipids, DiR, Bodipy FL-sphingomyelin, fluorescence microscopy

## Abstract

In this paper a rapid and highly efficient method for controlled incorporation of fluorescent lipids into living mammalian cells is introduced. Here, the fluorescent molecules have two consecutive functions: First, they trigger rapid membrane fusion between cellular plasma membranes and the lipid bilayers of their carrier particles, so called fusogenic liposomes, and second, after insertion into cellular membranes these molecules enable fluorescence imaging of cell membranes and membrane traffic processes. We tested the fluorescent derivatives of the following essential membrane lipids for membrane fusion: Ceramide, sphingomyelin, phosphocholine, phosphatidylinositol-bisphosphate, ganglioside, cholesterol, and cholesteryl ester. Our results show that all probed lipids could more efficiently be incorporated into the plasma membrane of living cells than by using other methods. Moreover, labeling occurred in a gentle manner under classical cell culture conditions reducing cellular stress responses. Staining procedures were monitored by fluorescence microscopy and it was observed that sphingolipids and cholesterol containing free hydroxyl groups exhibit a decreased distribution velocity as well as a longer persistence in the plasma membrane compared to lipids without hydroxyl groups like phospholipids or other artificial lipid analogs. After membrane staining, the fluorescent molecules were sorted into membranes of cell organelles according to their chemical properties and biological functions without any influence of the delivery system.

## 1. Introduction

Our understanding of biological membranes has expanded markedly within the last century [1,2]. Studies using fluorescently labeled membrane lipid components have contributed considerably to this progress. For example, they have been successfully used for identification and localization of cell compartments like endoplasmic reticulum, Golgi apparatus, endosomes or lysosomes [3,4,5,6,7]. Using fluorescent reporter molecules cellular events with lipid participation like lipid domain formation, disaggregation and re-organization [8,9,10,11,12,13,14], lipid internalization, trafficking and degradation [7,9,15,16,17,18], or protein-lipid interactions [16,19] have been monitored in the last years. Beside cell biology, biophysical studies have also applied some fluorescent lipids to characterize the physical properties of lipid bilayers [20,21], like membrane polarity, fluidity, lipid asymmetry, or their diffusion dynamics [19,22,23,24].

To be useful in such biological experiments a fluorescently labeled molecule has to meet two stringent requirements. First, the properties of the fluorescent label must be matched to fast and very sensitive detection in microscopy and spectroscopy, and second, the tagged molecule should be a true reporter, *i.e.*, its behavior must follow that of its untagged counterpart as closely as possible. These two expectations seem to be contradicting since fluorescent monitoring requires the presence of large aromatic moieties for excitation by visible light. Because natural membrane lipids do not have such molecular parts, their linkage to fluorescent groups, so called fluorophores, is essential to make them detectable by fluorescent microscopy. The most favored candidates for this purpose are fluorophores with high photostability, good molar absorptivities, high quantum yields, and emission maxima in the visible region, e.g., Bodipy-, Di-, or Atto-dyes. 

Once a fluorescently tagged lipid matching the experimental requirements has been found or synthesized it must be intercalated into the membranes of living cells which is often a surprisingly challenging endeavor. For this purpose several methods have been established and improved during the many decades of membrane research. The easiest but least effective way is incubation of living cells in a medium supplemented with fluorescent lipids. In this case, a small amount of labeled lipids is taken up by cellular endocytosis while other molecules are moved into or across the plasma membrane by energy-independent flippases [25]. For example, the fluorescent labeled ceramide derivate, Bodipy FL ceramide, can effectively be incorporated into cells in this way. It mostly accumulates in the Golgi apparatus, its primary accumulation site and to a lesser extent in the endoplasmic reticulum, its synthesis site [4,18]. Therefore this labeled lipid is widely used in cell biology studies for the identification and staining of these cellular compartments [3]. Some synthetic function-spacer-lipid constructs as well as artificial amphipathic molecules from the fluorescent Di-series, e.g., DiO and some DiIs, can also be easily and efficiently taken up from the cellular environment by the plasma membrane. Therefore they are used as plasma membrane staining molecules [26,27]. However, the structure of these molecules doesn’t mimic any natural membrane component. While the lipid flip-flop motion is an effective incorporation mechanism for some of the above mentioned lipids, it fails for most membrane lipids. Moreover, the efficiency of lipid uptake is in most cases low, resulting in weak fluorescent signals and problematic detection, correspondingly. 

Pagano and co-workers reported another technique in the early 80s to increase the lipid uptake [28]. His method rests on incubation of living cells with fluorescent lipids complexed to BSA at lower temperatures, e.g., 4 °C for 30 min, and a subsequent rapid temperature increase to 37 °C. The incubation at low temperature causes the activation of protective cell mechanisms and results in slowed down cell functions. With increasing temperatures cell functions are accelerated including the endocytotic uptake of fluorescent lipids. After lipid uptake, the fluorescent molecules are sorted into cell organelles according to their biological properties allowing microscopic imaging. Pagano’s pioneering work allowed for the first time a successful fluorescence monitoring of lipid trafficking in living cells. For all that, this method has also some drawbacks. For example, the incubation at low temperature causes cellular stress responses [29,30]. Moreover, the increased cell endocytotic uptake of molecules yields still low fluorescent signal intensities which renders fluorescence detection difficult.

**Figure 1:**
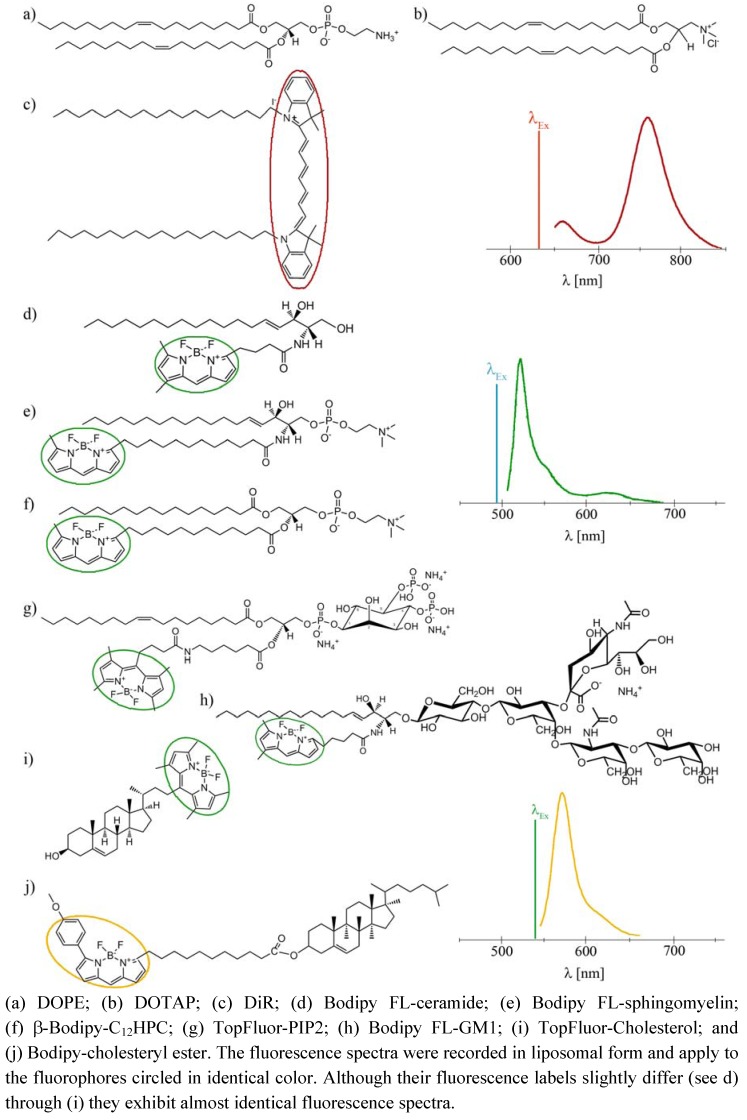
Molecular structures and fluorescence spectra of the lipid derivates used in this study.

To eliminate these drawbacks, a new method has been established for direct lipid insertion into the plasma membrane [31]. This technique is based on the observation that fluorescent lipids induce highly efficient and rapid membrane fusion between cellular plasma membranes and the lipid bilayers of their carrier particles containing neutral and positively charged lipids (Figure 1a,b). Liposomes of that kind are called fusogenic liposomes. By membrane fusion, the fluorescent lipid shell inserts into the plasma membrane immediately modifying its composition. Labelling takes place directly within the plasma membrane of living cells at classical cell culture conditions without any stress responses. In this method, the fluorescent molecules have two consecutive functions: First, they are essential for fusion triggering, which is not trivial. Second, after insertion into cellular membranes, these molecules allow the fluorescence imaging of cell membranes and membrane traffic processes.

However, although all conventional fluorescently labeled lipids we tested up to now are able to trigger membrane fusion processes, a minimum concentration (2.5–5 mol%) is required to achieve this effect. In some cases such high amounts of a labeled lipid molecule could completely falsify the intended membrane study, e.g., cholesterol strongly influences membrane stiffness, lipid miscibility and micro domain formation already at low amounts [32]. To tackle this problem we introduce here an improved lipid incorporation technique based on two different fluorescent lipids. One biologically irrelevant fluorescent component triggers the membrane fusion at a concentration of about 3 mol% while the amount of the second, biological active component, is only determined by the purpose of the study, e.g., the detection requirements of fluorescence imaging. In this study, fluorescently labeled ceramide, sphingomyelin, phosphocholine, phosphatidylinositol-bisphosphate, ganglioside, cholesterol, and cholesterol ester (see Figure 1d–j) have been delivered to the plasma membrane of living cells at different concentrations using fusogenic liposomes. Membrane fusion has been mainly triggered by DiR (Figure 1c), a biological irrelevant lipid analogue. Distributions of fluorophores have been monitored during fusion of liposomal and cellular membranes and the subsequent time period of up to two days.

## 2. Results

### 2.1. Identification of Cellular Organelles Using Organelle Specific Fluorescent Markers

**Figure 2:**
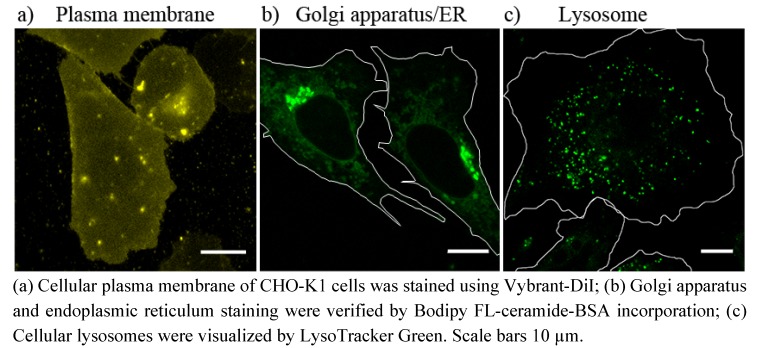
Identification of cellular organelles using organelle specific fluorescent markers.

Lipid trafficking between cell organelles is essential for various cell functions. However, before application of our new lipid delivery system for the analysis of such cellular lipid transport processes, putative target organelles had to be identified. For this purpose cellular organelles were visualized by fluorescence staining procedures known from literature (see the Experimental section). Plasma membranes of CHO-K1 cells were labeled using the fluorescent lipid analogue Vybrant-DiI. After incubation with Vybrant-DiI the whole cell surface exhibited yellow fluorescence (Figure 2a). The Golgi apparatus and the endoplasmic reticulum were identified by addition of Bodipy FL-ceramide-BSA complex to the cell culture. Figure 2b shows an intensive green signal around the nucleus, the Golgi apparatus and a less intense filamentous signal, the endoplasmic reticulum. Cellular lysosomes were stained with LysoTracker Green. Its green signal appeared in the whole cell body (except the nucleus) as small dots slowly moving in the cytoplasm (Figure 2c). Cellular distributions of these fluorescent marker molecules were subsequently compared with the signal distribution of lipids delivered by fusogenic liposomes. It should be mentioned here, that beside lysosomes, other cytoplasmic vesicular structures like endosomes or trafficking vesicles display nearly similar staining. Their explicit identifications were not achieved within this study therefore all stained vesicular compartments regardless of their exact identities were defined as lysosome like structures.

### 2.2. Incorporation of Bodipy FL-Sphingomyelin and DiR into Cardiac Fibroblast Using Fusogenic Liposomes

**Figure 3:**
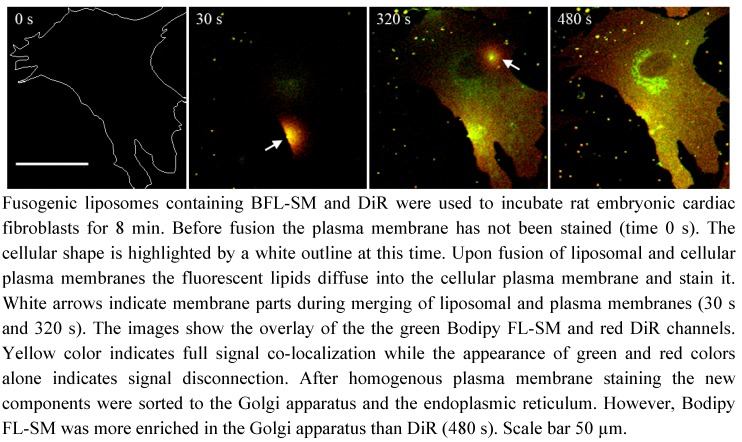
Plasma membrane staining with fusogenic liposomes.

To demonstrate the staining ability of fusogenic liposomes and to temporarily resolve the fusion process, the plasma membranes of rat embryonic cardiac fibroblasts were stained using such liposomes containing the artificial fluorescent lipid DiR (Figure 1c) and the chain labeled sphingomyelin derivate Bodipy FL-SM (Figure 1e) (DOPE/DOTAP/DiR/Bodipy FL-SM = 1/1/0.05/0.05 w/w). The fusion process was recorded by confocal laser scanning microscopy (see also Supplemental Movie 1). The advantage of using cardiac fibroblasts was their distinct flat cell body, which allowed simultaneous recording of upper and lower plasma membrane. Before vesicle addition (Figure 3: 0 s) no fluorescent signal was detected. Few seconds later, when the vesicles sedimented onto the cell surface, small yellow dots, green in the Bodipy FL-SM channel and red in the DiR channel, were detected. After membrane contact fluorescently labeled cell membranes appeared. This process was repeated several times during the observation time window of 8 min reaching a complete membrane staining (see also Supplemental Movie 1). Interestingly, the two fluorescent lipids did not fully co-localize with DiR distributing faster in the plasma membrane than Bodipy FL-SM (Figure 3: 30 s). However, these differences disappeared after a few minutes (Figure 3: 320 s) and the plasma membranes were homogenously labeled by both fluorescent molecules (compare Figure 2a and Figure 3: 320 s). Simultaneously with the homogenously stained plasma membrane the Golgi apparatus was saturated by Bodipy FL-SM while the endoplasmic reticulum contained both molecules (Figure 3: 480 s, see also Figure 2a,b). The analyzed cells showed no morphological changes during and after vesicle fusion. Cells remained adherent to the substrate and no membrane blebbing occurred. Even cell shape remained unaffected during the observation time of up to several hours.

### 2.3. Cellular Distribution of Bodipy FL-Sphingomyelin and DiR in CHO-K1 Cells after Incorporation via Fusogenic Liposomes

**Figure 4:**
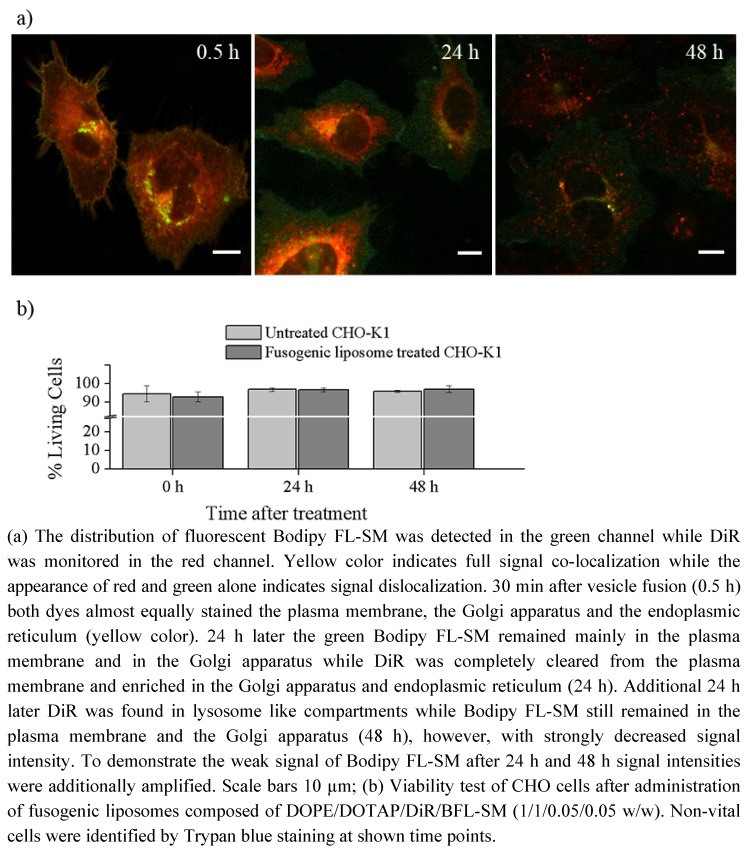
Cellular distribution of Bodipy FL-SM and DiR in CHO-K1 cells after incorporation via fusogenic liposomes.

To follow the time dependent signal distribution of the chain labeled sphingomyelin, Bodipy FL-SM, and the lipid analogue DiR they were incorporated into CHO-K1 cells using fusogenic liposomes and the signal distributions were monitored over 48 h. The other cell type was chosen to demonstrate the ubiquitous staining ability of the liposomes. 30 min after vesicle fusion the main part of the green fluorescence signal of sphingomyelin was distributed between plasma membrane and Golgi apparatus, with a smaller fraction localized in the endoplasmic reticulum (Figure 4: 0.5 h). A similar sphingomyelin distribution was also observed in cardiac fibroblasts immediately after lipid delivery (Figure 3: 480 s). This signal distribution could be detected over 48 h, however the signal intensity immensely decreased over this time period (Figure 4: 24 h and 48 h) due to cell proliferation.

Simultaneously with Bodipy FL-SM, DiR was also intercalated into the plasma membrane of CHO-K1 cells and its red fluorescence was monitored. After an intense plasma membrane–endoplasmic reticulum–Golgi apparatus staining (Figure 4: 0.5 h) DiR was rapidly cleared from the plasma membrane and the first labeled lysosome like compartments appeared 24 h after intercalation (Figure 4: 24 h). Additional 24 h later some weak endoplasmic reticulum and strong lysosome like staining were observed (compare Figure 4: 48 h and Figure 2c).

Viability assays were also carried out over 48 h after liposome administration to test staining toxicity over time. As Figure 4b shows, cell viability after staining with fusogenic liposomes was comparable with that of untreated cells (between 94 and 97%) and even total cell numbers did not differ (data not shown).

### 2.4. Comparison of Lipid Incorporation Using Fusogenic Liposomes and BSA Complexes

**Figure 5:**
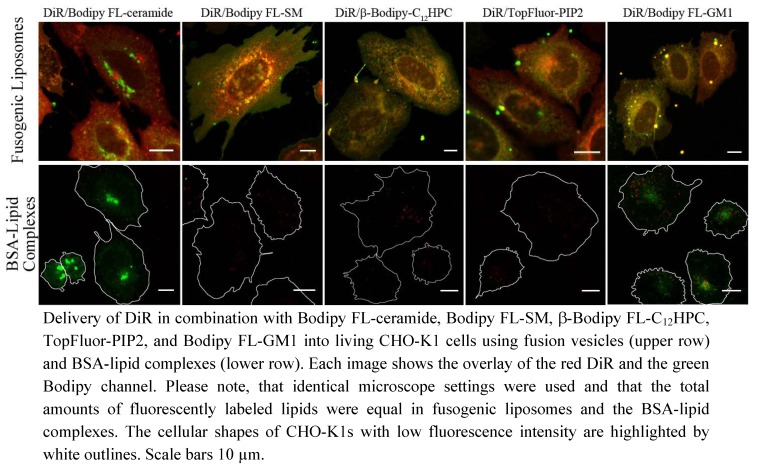
Comparison of lipid incorporation using fusogenic liposomes and BSA complexes.

To demonstrate the increased staining ability of fusogenic vesicles compared to other techniques, CHO-K1 cells were incubated with fusogenic liposomes at 37 °C for 10 minutes or with fluorescent lipids complexed with bovine serum albumin (BSA) protein at 4 °C for 30 min and subsequently warmed up to 37 °C and incubated for further 30 min as described by Lipsky *et al.* [28]. As shown in Figure 5 all probed lipids, the chain labeled ceramide (Figure 1d), sphingomyelin (Figure 1e), phosphocholine (Figure 1f), phosphatidylinositol-bisphosphate (Figure 1g), ganglioside (Figure 1h), simultaneously with the lipid analog DiR (Figure 1c) (DOPE/DOTAP/DiR/fluorescent lipid = 1/1/0.05/0.05 w/w) were successfully incorporated into the cellular plasma membrane by incubation with fusogenic liposomes (Figure 5: Fusogenic Liposomes). The fluorescence signals of all used lipids were adequate for microscopy. Cell shape and morphology of CHO-K1 cells remained uninfluenced during and after membrane fusion.

Using the same amount of lipids like above but complexed to BSA all probed molecules showed weak membrane intercalation and were mainly localized in endosomes/lysosome like compartments with the exception of ganglioside and ceramide (Figure 5: BSA-complexes). These reached the Golgi apparatus at high concentration and the endoplasmic reticulum at lower concentration. In some cases a weak endocytotic incorporation of DiR was also observed (red dots in Figure 5: BSA-Lipid Complexes). Parallel to the overall low fluorescent intensity, almost all cells showed obvious membrane blebbing (see the pronged white outlines) and had a rather roundish cell shape compared to cell morphology before incubation with lipid-BSA complexes. This stress response was most likely induced by the cold shock and disappeared after some hours.

### 2.5. Incorporation of Fluorescent Cholesterol Derivates into Cardiac Fibroblasts Using Fusogenic Liposomes

**Figure 6:**
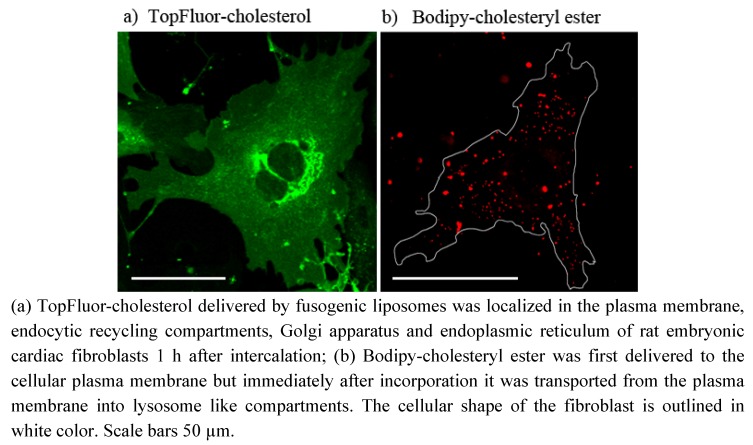
Incorporation of cholesterol derivatives into cardiac fibroblasts using fusogenic liposomes.

Besides phospho- and glycolipids two different fluorescent cholesterol derivatives were also delivered to rat embryonic cardiac fibroblasts using fusogenic liposomes. The fluorescent cholesterol derivate TopFluor-cholesterol (Figure 1i) was mixed to the fusogenic lipid mixture in a concentration of DOPE/DOTAP/DiR/TopFluor-cholesterol = 1/1/0.05/0.025 w/w. Immediately after membrane fusion a green, homogenous plasma membrane staining was detected. Some minutes later the fluorescent cholesterol signal was distributed between plasma membrane, endoplasmic reticulum, Golgi apparatus and vesicular compartments (Figure 6a). These cholesterol-stained small and finely dispersed structures seemingly differed from those stained by LysoTracker Green (Figure 2c). The fluorescence signal of DiR was collected in the red channel but not shown in Figure 6a to avoid obscuring the green signal.

The esterified fluorescent cholesterol analog, Bodipy-cholesteryl ester (Figure 1j), was also incorporated into the plasma membrane of fibroblasts by DOPE/DOTAP/Bodipy-cholesteryl ester = 1/1/0.1 w/w liposomes. In this case membrane fusion was induced by the cholesterol derivative itself. Because Förster resonance energy transfer (FRET) between Bodipy and DiR would completely quench the fluorescence signal of the cholesteryl ester we relinquish to use DiR for the exact cellular localization of Bodipy-cholesteryl ester. Shortly after plasma membrane staining, the fluorescent signal disappeared from the membrane and was localized in lysosome like compartments (Figure 6b). 

## 3. Discussion

Fluorescently labeled membrane lipids are powerful tools for biological membrane studies with microscopic techniques. For these purposes, they have to meet the following needs: The molecules have to authentically mimic the properties of membrane components even after coupling of fluorophore and natural lipid. After labeling, an appropriate method is needed for the incorporation of the chemically modified membrane components into living cells and their membranes. And finally, they have to be sensitively detected by fluorescence microscopic techniques.

In this study, a new method has been developed for effective fluorescent lipid incorporation into cellular membrane systems using commercial fluorescently labeled membrane lipids. Our system is based on positively charged phospholipid vesicles containing fluorescently labeled lipids or similar amphipathic molecules in a concentration of 2–5 mol%. This concentration of fluorophores containing delocalized electrons is sufficient to interact with the strong positive charges in the head group region of liposomes inducing temporal dipoles. These dipoles presumably yield local disorders of molecular arrangements in the bilayer and trigger membrane fusion processes with the plasma membrane as we proposed earlier [31]. Here, the determining factors are the high positive charge of liposomes as well as the molar ratio between positively charged and neutral lipids and aromatic compounds. After fusion, the liposome membranes rapidly merge with the cellular plasma membrane and make the latter visible for fluorescence microscopy. However, although only the fluorescent lipid is detected we assume that all lipid components of the vesicles are delivered into the plasma membrane. This statement is supported by the results on vesicles containing more than one fluorescent membrane of different mammalian cell types using positively charged lipid. In this work, DiR, a lipid mimicking amphipathic fluorophore together with sphingomyelin, a natural membrane component labeled by Bodipy FL, have been successfully incorporated in one step into the plasma vesicles (Figure 3 and Figure 4). We also tested numerous other dye combinations like phospholipids, other sphingo- and glycolipids (see Figure 5), cholesterol (Figure 6) in combination with DiR or other members of the Di series or phospholipids combined with shingolipids (data not shown). Our results show that all labeled components successfully stained the plasma membrane in combination with each other. The main advantage of such combined fusogenic particles is the controlled delivery of fluorescent molecules in a broad concentration range. For example, one biologically irrelevant fluorescent component, e.g., DiR, can trigger the membrane fusion at a higher concentration, while the amount of the second, biological active component is freely adjustable.

The distribution of the extrinsic fluorescent component in the cellular plasma membrane indicated a multi component chemical interplay in the membrane bilayer between the new molecules and cellular membrane components. For example, lipids containing free hydroxyl groups like sphingolipids or cholesterol interact more strongly with other membrane components by hydrogen bonds than phospholipids, respectively [10]. These interactions could noticeably decrease the distribution velocity of these lipids in the new bilayer compared to molecules without hydroxyl groups like phospholipids or the members of the Di-series as we have observed (see Figure 3). Even on a nanoscopic scale, the strong interaction of sphingolipid analogs with other membrane components due to transient hydrogen bonding was proven by Mueller *et al.*, whereas phospholipid analogs revealed weak interactions [33]. This molecular character influences the membrane persistence of new molecules on the short time scale, like during merging of vesicular and plasma membranes and subsequent lateral lipid mixing. In addition to this, lipids containing free hydroxyl groups show much longer plasma membrane localization on long time scales (e.g., over 48 h) than lipids lacking these hydroxyl groups. For example, comparing the membrane persistence of sphingomyelin and DiR, we have observed that sphingomyelin is located over several days in the plasma membrane while DiR is cleared from the plasma membrane in some hours (Figure 4). Although the fluorescent components enabled the observation of the molecular localization for such a long time, it should be kept in mind that there are several processes working in the cellular metabolism which could gravely change the originally delivered molecules during the observation period affecting our conclusions [18]. However, this systematic error would not only influence our method but also all other measurements and techniques applying fluorescent lipid derivates.

After lipid merging, cellular mechanisms need distinct time periods to recognize the new components and to sort them according to their biological function and chemical properties. If the fluorescent labeling or the delivery system does not gravely change the chemical behavior of the original molecule it will be transported to its cellular destination after successful recognition. For example, the chain labeled ceramide delivered by fusogenic liposomes has been mainly accumulated in the Golgi apparatus, its primary accumulation site and in the endoplasmic reticulum, its synthesis site (Figure 4) [4,18]. It should be mentioned here that due to its high hydrophobicity this lipid is rapidly internalized from the environment even without any delivery system [3]. In this case, it is also mainly enriched in the Golgi apparatus and it slightly stains the endoplasmic reticulum, similar to our case or to the BSA-complexed delivery (Figure 4). Therefore we also used this fluorescent ceramide for the identification of the Golgi apparatus and endoplasmic reticulum.

Although sphingomyelin or ganglioside are also sphingolipids, they strongly differ from ceramide in their head group region (Figure 1d,e,h) which results in low lipid uptake without a delivery system. Using positively charged delivery vesicles the chain labeled sphingomyelin could be directly inserted into the plasma membrane of different cell types. The main part of this signal remained in the plasma membrane over 48 h while the other part was identified in the Golgi apparatus (Figure 4) according to the cellular occurrence of sphingomyelin [18,34]. Based on its high hydrophobicity and the fact that, unlike ceramide, sphingomyelin cannot overcome the plasma membrane barrier without delivery system to reach the Golgi apparatus, we assume that the rapid transport of Bodipy FL-SM between plasma membrane and Golgi apparatus is due to sphingomyelin-transporter proteins [18]. It should be also mentioned here, that our delivery method is a gentle process for living cells contrarily to the lipid delivery by BSA-lipid-complexes. It is known from literature that sphingolipid traffic is strongly influenced by stress factors [18]. Therefore avoiding environmental stress is a promising strategy in general and especially for studies focusing on sphingolipids. 

Beside sphingolipids some phospholipid derivates have also been probed for efficient cellular incorporation using fusogenic liposomes. The chain labeled phosphocholine first accumulated in the plasma membrane then was rapidly transferred to the Golgi apparatus [6] and the endoplasmic reticulum [35] as described by authors cited above. We suggest that this lipid was recognized and subsequently transported by phospholipid specific transporters [36]. Similar to phosphocholine the fluorescent derivate of the signal molecule phosphatidylinositol-bisphosphate has also been recognized by the cellular machinery and has been transported from the plasma membrane to the endoplasmic reticulum, presumably by specific phosphatidylinositol transfer proteins [37].

To demonstrate the ubiquitous ability of our fusogenic liposomes two fluorescent derivates of cholesterol have also been delivered to the plasma membrane of mammalian cells. One of them is labeled on the short side chain by Bodipy FL (see Figure 1i). This dye exhibits a relatively hydrophobic character and a small size. However, the fluorophore and cholesterol together are more than 50% larger than cholesterol alone. For all that, we found a similar distribution of TopFluor-cholesterol delivered by fusogenic vesicles as Hao *et al.* [17] for dehydroergosterol, the presumably best cholesterol analog. He observed an ergosterol distributed between plasma membrane, its accumulation place, endocytic recycling compartments, its transport system, and endoplasmic reticulum, its synthesis organelle. We could also identify the fluorescent signal of our cholesterol derivate in the plasma membrane as well as in the endoplasmic reticulum. The additional cytoplasmic vesicular signal could be rather assigned to endocytic recycling compartments then to lysosomes (compare Figure 2c and Figure 6a). As described by Hao *et al.* ergosterol is enriched in endocytic recycling compartments by transport processes between plasma membrane and Golgi apparatus [17]. Based on the similarities between ergosterol and TopFluor-cholesterol we assume that TopFluor-cholesterol stained also such compartments.

The other cholesterol derivate is esterified on its hydroxyl-group by a fatty acid (C11) attached to a larger Bodipy-fluorophore (Figure 1j). The biological function of such cholesteryl ester is the storage of excess cellular cholesterol. Based on its conical molecular shape it doesn’t fit into the flat phospholipid bilayer but rather builds some droplets, so called cytoplasmic lipid droplets [15]. However, the Bodipy-labeling on the cholesteryl ester used in this study drastically changed the natural conical molecular shape to a rather dumbbell-shape, its cellular behavior doesn´t differ from that of its natural analog. As we observed Bodipy-cholesteryl ester was transported from the plasma membrane immediately after incorporation into lysosome like compartments, presumably into cytoplasmic lipid droplets indicating strong preference for droplet structures instead of flat bilayers.

These data support our suggestion that the delivery system itself doesn´t influence the distribution behavior of the delivered fluorescent molecules. They can freely move in cellular membranes and their traffics are controlled by cellular processes.

## 4. Experimental

### 4.1. Materials

1,2-Dioleoyl-*sn*-glycero-3-phosphoethanolamine (DOPE), 1,2-dioleoyl-3-trimethylammonium-propane, chloride salt (DOTAP), 1-oleoyl-2-{6-[4-(dipyrrometheneboron difluoride)butanoyl]amino}hexanoyl-*sn*-glycero-3-phosphoinositol-4,5-bisphosphate ammonium salt (TopFluor-PIP2), and 23-(dipyrro-metheneboron difluoride)-24-norcholesterol (TopFluor-cholesterol) were purchased from Avanti Polar Lipids, Inc. (Alabaster, AL, USA). All other fluorescent lipids 1,1'-dioctadecyl-3,3,3',3'-tetramethylindotricarbocyanine iodide (“DiR”; DiIC_18_(7)), 2-(4,4-difluoro-5-methyl-4-bora-3*a*,4*a*-diaza*s*-indacene-3-dodecanoyl)-1-hexadecanoyl-*sn*-glycero-3-phosphocholine (β-Bodipy FL-C_12_HPC), *N*-(4,4-difluoro-5,7-dimethyl-4-bora-3a,4a-diaza-*s*-indacene-3-pentanoyl) sphingosine (Bodipy FL-ceramide), *N*-(4,4-difluoro-5,7-dimethyl-4-bora-3a,4a-diaza-*s*-indacene-3-dodecanoyl)sphingosyl phosphocholine (Bodipy FL-SM), *N*-(4,4-difluoro-5,7-dimethyl-4-bora-3a,4a-diaza-*s*-indacene-3-dodecanoyl)ganglioside (Bodipy FL-GM1), and cholesterol 4,4-difluoro-5-(4-methoxyphenyl)-4-bora-3a,4a-diaza-*s*-indacene-3-undecanoate (Bodipy-cholesteryl ester) were ordered from Invitrogen (Eugene, OR, USA). The chemical structures of fluorescent lipids used in this study are shown in Figure 1a–j.

### 4.2. Preparation of Fusogenic Liposomes

Lipid components like DOPE, DOTAP, and the fluorescent lipids were mixed in chloroform in a weight ratio of DOPE/DOTAP/fluorescent lipid of 1/1/0.05–0.1. Chloroform was evaporated under vacuum for 0.5–1 h. Then, lipids were dispersed in 20 mM 2-(4-(2-hydroxyethyl)-1-piperazinyl)-ethansulfonic acid (HEPES) buffer (VWR, Darmstadt, Germany) at a total lipid concentration of 2.1 mg/mL. The solution was vortexed for approximately 1–2 min to produce multilamellar liposomes. After homogenization in an ultrasonic bath for 10–20 min, mainly unilamellar vesicles or liposomes were formed.

For fusion experiments, liposome stock solution (10 μL) was diluted 1/100 with appropriate cell culture medium (see cell culture) and gently shaken for 1–2 min at room temperature. Cells in a Petri dish (Ø = 3.5 cm), were incubated in fusogenic liposomes solution (pH 7.4, 1 mL) for 5–15 min at 37 °C. Subsequently, the fusion mixture was replaced by fresh medium.

### 4.3. Preparation of Lipid-Bovine Serum Albumin (BSA) Complexes

One hundred µM fluorescent lipid stock solution (25 µL) and 1 mM DiR stock solution (2.5 µL) were dispensed into a small glass test tube and dried under vacuum for at least 1 h. The dried molecules were then dissolved in absolute ethanol (200 µL). Meanwhile, defatted BSA (3.4 mg, Merck KGaA, Darmstadt, Germany) was added to a HBSS/HEPES solution (10 mL, Hanks buffered salt solution and 10 mM HEPES, pH 7.4). The lipid-ethanol solution (200 µL) was then injected into the HBSS/HEPES solution (10 mL) while vortexing. This resulted in a final concentration of 250 nM fluorescent lipid, 250 nM DiR, 5 µM BSA and 2% (v/v) ethanol. For cell staining, cells were incubated with lipid-BSA complex solution (2 mL) on ice for 30 min. Afterwards, cells were rinsed with cold PBS buffer and incubated in fresh cell culture medium for an additional 4 min at 37 °C before imaging began. Please note, that the total amount of fluorescent lipids added via lipid-BSA complexes was equal to the total amount added via fusogenic liposomes.

### 4.4. Cell Culture

#### 4.4.1. Cardiac Fibroblasts

Cardiac fibroblasts were isolated from 18-day old Wistar rat embryos as described earlier [38]. Cells were seeded on fibronectin coated glass surfaces [2.5 μg/cm^2^ human plasma fibronectin (BD Biosciences, San Jose, CA, USA)]. Cells were maintained in F10 Ham’s medium (Sigma-Aldrich, St. Louis, MO, USA) supplemented with 10% fetal bovine serum, a 1/100 dilution of an antibiotic solution [10,000 units penicillin and 10 mg/mL streptomycin in 0.9% NaCl, (Sigma-Aldrich)] and a 1/200 dilution of solution containing insulin (1 mg/mL), transferrin (0.55 mg/mL) and sodium selenite (0.5 µg/mL) in Earle’s balanced salt solution (EBSS) (Sigma-Aldrich). During culture as well as experimental steps, cells were kept at 37 °C and 5% CO_2_ in a saturated humid atmosphere. For liposome fusion and microscopy, 3,000 cells were seeded on fibronectin coated glass surfaces (2.5 μg/cm^2^ human plasma fibronectin (BD Biosciences) two days prior to the experiment.

#### 4.4.2. Chinese Hamster Ovary K1 Cells (CHO-K1)

CHO-K1 cells were purchased from American Type Culture Collection (ATTC, Manassas, VA, USA). They were maintained in DMEM-F12 (Sigma-Aldrich) supplemented with 10% fetal bovine serum and a 1/100 dilution of an antibiotic solution [10,000 units penicillin and 10 mg/mL streptomycin in 0.9% NaCl, (Sigma-Aldrich)]. During culture as well as experimental steps, cells were kept at 37 °C and 5% CO_2_ in a saturated humid atmosphere. Cell density never exceeded 80% confluence. For liposome fusion and microscopy, 6,000 cells were seeded on fibronectin coated glass surfaces (2.5 μg/cm^2^ human plasma fibronectin, BD Biosciences) two days prior to the experiment. 

#### 4.4.3. Cell Viability Test

7.5 × 10^4^ CHO-K1 cells were seeded on small (Ø = 3.5 cm) cell culture dishes (Greiner, Solingen, Germany) one day prior to the experiment and maintained as described above. At the next day cell culture dishes with CHO-K1 cells at a confluency of approx. 40% were then treated with fusogenic liposomes containing DOPE/DOTAP/DiR/Bodipy FL-SM = 1/1/0.05/0.05 w/w as described in Section 4.2. After membrane labeling, cells were incubated for 10 further minutes to allow proper distribution of the added lipids. Immediately, 24 h and 48 h after staining, cells were incubated with a 0.5% Trypsin/0.2% EDTA solution (1 mL, Sigma) for 3 min at 37 °C and collected by centrifugation at 500 g for 3 min. The pellet was resuspended in phosphate buffered saline (PBS, pH 7.4). The cell suspension (20 µL) was then incubated with a 0.5% Trypan Blue (Sigma)/0.9% NaCl solution (80 µL) for 2 min at 37 °C. Afterwards, the relative amount of living cells was determined in a Neubauer counting chamber. The rate of living cells after membrane fusion was compared with an untreated control. Every time point was analyzed 5 times in independent experiments.

### 4.5. Identification of Cellular Organelles Using Organelle Specific Fluorescent Markers

#### 4.5.1. Plasma Membrane Staining

Plasma membrane of CHO-K1 cells was stained using the lipophilic tracer molecule Vybrant-DiI (Invitrogen) [39]. Cells were incubated at 37 °C with 5 µM Vybrant-DiI in DMEM-F12 for 5 min, washed once with PBS and normal cell culture medium was added. Cells were imaged with a fluorescence microscope immediately after staining, so that no intracellular membrane staining occurred.

#### 4.5.2. Golgi Apparatus/Endoplasmic Reticulum Staining

Golgi apparatus was stained using the method established by Lipsky *et al.* [27]. For that purpose, CHO-K1 cells, grown on glass cover slips, were incubated for 30 min. at 4 °C with 5 µM Bodipy FL-ceramide-BSA in PBS [see “Preparation of lipid-bovine serum albumin (BSA) complexes”]. Afterwards, cells were rinsed several times with ice-cold PBS and incubated in fresh cell culture medium at 37 °C for additional 30 min. The samples were then washed with fresh medium and examined using a fluorescence microscope.

#### 4.5.3. Lysosomes Staining

Lysosomes were stained using the LysoTracker Green DND-26 (Invitrogen) [40]. CHO-K1 cells, grown on glass cover slips, were incubated with 50 nM of the LysoTracker, dissolved in cell culture medium, at 37 °C for at least 30 min. The LysoTrackr-containing medium was then replaced by fresh cell culture medium and samples were imaged using a fluorescence microscope. 

### 4.6. Fluorescence Microscopy

Samples were imaged using a laser scanning microscope LSM 710 (Carl Zeiss MicroImaging GmbH, Jena, Germany) equipped with an argon ion laser (488 nm), a green helium neon laser (543 nm) and a red helium-neon laser (633 nm). To detect the fluorescent signal of BODIPY FL (ex. 488 nm), a band pass filter BP 500–550 nm, for BODIPY TMR (ex. 543 nm) a long pass filter LP 560 nm and for DiR (ex. 633 nm) also a long pass filter LP 650 nm was used. The emission spectra of these two dyes do not overlap. No FRET occurs between these two dyes. For the cell organelle stainings, LysoTracker Green DND-26 and Bodipy FL ceramide were excited at 488 nm and fluorescence signals collected using band pass filter BP 500–550 nm. DiI was excited at 543 nm and the fluorescence signal was collected using BP 550–600 nm. To avoid detection of autofluorescence background signals, laser intensities never exceeded 4% for the argon ion- and 10% for the helium-neon laser, respectively. The microscope was equipped with an oil immersion objective EC Plan-Neofluar 40×/1.30 Ph3 or a water immersion objective 40×/1.20 C-Apochromat (both from Carl Zeiss). To maintain appropriate culture conditions during experimental steps, the microscope was equipped with an incubator (Incubator XL 2, Carl Zeiss) and temperature as well as CO_2_ was kept at 37 °C and 5%, respectively. The images were analyzed with the LSM 710 ZEN software (Carl Zeiss).

### 4.7. Fluorescence Spectroscopy

The fluorescent spectra of the labeled lipids used in this study were recorded by a fluorescence spectrometer (Fluorolog-3, HORIBA Jobin Yvon, Edison NJ, USA). Lipids were prepared in liposomal form mixed to 1,2-dioleoyl-sn-glycero-phosphocholine (DOPC) in a molar ratio of DOPC/DiR, DOPC/β-Bodipy FL-C_12_HPC and DOPC/Bodipy-cholesteryl ester 200:1 mol/mol. We assume that the fluorescent spectra of the different Bodipy derivates, e.g., β-Bodipy FL, Bodipy FL, and TopFluor do not differ significantly. The total lipid concentration was adjusted to 2 mg/mL in 20 mM Hepes buffer. Recording conditions like excitation and detection wavelengths were chosen similar to microscopical parameters.

## 5. Conclusions

The here introduced delivery method of fluorescent lipids to the plasma membrane of living cells is a rapid, simultaneously effective and gentle tool for controlled cellular membrane staining by almost arbitrary lipid molecules and for subsequent molecular traffic analysis. The fusion process between liposomal and cellular membranes appears to be a general one without cell type or lipid dependence. The intercalated components can immediately be monitored via their fluorescence over a period of up to 2 days without any perceptible cellular damages. This technique could also be useful for delivery of other fluorescently labeled membrane components e.g., proteins to living cells.

## Supplementary Materials

Supplementary File 1
